# HLA alleles associated with asparaginase hypersensitivity in Chinese children

**DOI:** 10.1186/s13045-021-01201-3

**Published:** 2021-10-30

**Authors:** Gilbert T. Chua, Jaime S. Rosa Duque, Daniel Ka Leung Cheuk, Alex Wing Kwan Leung, Wilfred Hing Sang Wong, Anthony Pak Yin Liu, Pamela P. W. Lee, Shau Yin Ha, Alan Kwok Shing Chiang, Marco Hok Kung Ho, Wai Keung Chu, Yuk Sing Chan, Chun Wing Luk, Alvin Siu Cheung Ling, Mike Yat Wah Kwan, Oscar Kuen Fong Yiu, Ian Chi Kei Wong, Yu Lung Lau, Chi Kong Li, Wing Hang Leung, Godfrey Chi Fung Chan, Patrick Ip, Janette Kwok

**Affiliations:** 1 Department of Paediatrics and Adolescent Medicine, Li Ka Shing Faculty of Medicine, 1/F, New Clinical Building, Queen Mary Hospital, The University of Hong Kong, 102 Pokfulam Road, Pokfulam, Hong Kong SAR China; 2 Department of Paediatrics and Adolescent Medicine, Hong Kong Children’s Hospital, Kowloon, Hong Kong SAR China; 3 Department of Paediatrics, The Chinese University of Hong Kong, Shatin, Hong Kong SAR China; 4 Division of Transplantation and Immunogenetics, Department of Pathology, Queen Mary Hospital, 15/F, Block T102 Pokfulam Road, Pokfulam, Hong Kong SAR China; 5 Department of Paediatrics and Adolescent Medicine, Princess Margaret Hospital, Kwai Chung, Hong Kong SAR China; 6 Laboratory of Data Discovery for Health (D24H), Hong Kong Science and Technology Park, Shatin, Hong Kong SAR China; 7 Centre for Safe Medication Practice and Research, Department of Pharmacology and Pharmacy, The University of Hong Kong, Pokfulam, Hong Kong SAR China; 8 Research Department of Practice and Policy, UCL School of Pharmacy, University College, London, UK

**Keywords:** Human leukocyte antigens, Asparaginase, Hypersensitivity, Allergy, Chinese, Children

## Abstract

Asparaginase is an important drug to treat childhood haematological malignancies. Data on the association between human leukocyte antigens (HLA) and asparaginase hypersensitivity among Chinese are lacking. We conducted a retrospective study to identify HLA alleles associated with asparaginase hypersensitivity among Chinese children with acute lymphoblastic leukaemia (ALL), mixed phenotype leukaemia and non-Hodgkin lymphoma (NHL), who received asparaginases with HLA typing performed between 2009 and 2019. 107 Chinese patients were analysed. 66.3% (71/107) developed hypersensitivity to at least one of the asparaginases. HLA-B*46:01 (OR 3.8, 95% CI 1.4–10.1, *p* < 0.01) and DRB1*09:01 (OR 4.3, 95% CI 1.6–11.4, *p* < 0.01) were significantly associated with l-asparaginase hypersensitivities, which remained significant after adjustment for age, gender and B cell ALL [HLA-B*46:01 (adjusted OR 3.5, 95% 1.3–10.5, *p* = 0.02) and DRB1*09:01 (OR 4.4, 95% CI 1.6–13.3, *p* < 0.01)].

**Supplementary Information:**

The online version contains supplementary material available at 10.1186/s13045-021-01201-3.

## To the editor:

Asparaginase is one of the essential drugs for the curative treatment of acute lymphoblastic leukaemia (ALL) [1]. About 30–75% of patients receiving native *Escherichia coli* asparaginase experience hypersensitivity reactions [1, 2]. Furthermore, patients who were inadequately treated with asparaginase because of hypersensitivity reactions have worse outcomes compared with patients with fewer reactions that can tolerate more asparaginase doses [3]. Recent studies involving patients with European ancestry showed that the rs6021191 variant in NFATC2 and the rs17885382 in HLA-DRB1 were associated with an increased risk of asparaginase hypersensitivity [4, 5]. Nevertheless, data from the Chinese populations are lacking.

Between 1 January 2009 and 31 December 2019, 107 Chinese patients with ALL, mixed phenotype leukaemia and non-Hodgkin lymphoma (NHL), who received asparaginases with HLA typing performed were analysed. The detailed study methodology is described in Additional file 1. There were no significant differences in terms of the demographics, primary diagnoses and outcomes of the patients with and without asparaginase hypersensitivity (Table 1). The distribution of asparaginase exposure, hypersensitivity and anaphylaxis to l-asparaginase, peg-asparaginase and erwinase is illustrated in Additional file 1: Table S1. None of them developed the reactions on the first dose of injection.

**Table 1:** Comparison of demographics, diagnoses and outcomes between patients with and without asparaginase hypersensitivity to at least one form of asparaginase

	Asparaginase hypersensitivity *N* = 71	No asparaginase hypersensitivity *N* = 36	*p* values
*Demographics*			
Median age at diagnosis (years, interquartile ranges)	4.6 (2.2–12.0)	6.1 (2.1–14.1)	0.53
Male (%)	47 (66.2)	22 (61.1)	0.67
Other allergies^#^	10 (14.8)	1 (2.8)	0.09
Drug hypersensitivity	6	1	–
Asthma	2	0	–
Eczema	2	0	–
Allergic rhinitis	2	0	–
Food allergy	1	0	–
*Diagnoses*			
B lineage ALL (%)	54 (76.1)	23 (63.9)	0.25
B cell ALL (%)	46 (64.8)	16 (44.4)	0.06
Infant ALL (%)	5 (7.0)	5 (13.9)	0.30
Ph + ALL (%)	3 (4.2)	2 (5.6)	0.99
T cell ALL (%)	10 (14.1)	7 (19.4)	0.58
Mixed phenotype acute leukaemia (%)	2 (2.8)	4 (11.1)	0.18
T cell lymphoma (%)	5 (7.0)	2 (5.6)	0.99
*Outcomes*			
Disease relapse (%)	37 (52.1)	18 (50.0)	0.84
BMT (%)	34 (47.9)	16 (44.4)	0.84
Death (%)	22 (31.0)	13 (36.1)	0.66

ALL, acute lymphoblastic leukaemia; BMT, bone marrow transplantation; Ph + , Philadelphia chromosome

^#^Some patients have multiple allergic conditions

HLA-B*51:01 was enriched among children with at least one asparaginase hypersensitivity, and HLA-B*46:01, DRB1*09:01 and B*51:01 were enriched among children l-asparaginase allergies (Additional file 1). HLA-B*46:01 (OR 3.8, 95% CI 1.4–10.1, *p* < 0.01) and DRB1*09:01 (OR 4.3, 95% CI 1.6–11.4, *p* < 0.01) were significantly associated with l-asparaginase hypersensitivity. Multiple logistic regression showed that HLA-B*46:01 (adjusted OR 3.5, 95% CI 1.3–10.5, *p* = 0.02) and DRB1*09:01 (adjusted OR 4.4, 95% CI 1.6–13.3, *p* < 0.01) remained significantly associated with l-asparaginase allergy after adjusting for age, gender and the diagnosis of B cell ALL. HLA-B*51:01 was not associated with any asparaginase hypersensitivities (Table 2). Of note, 19 patients carried both HLA-B*46:01 and DRB1*09:01. 18 of them (94.7%) had l-asparaginase hypersensitivity, and 10 of them (52.6%) also had peg-asparaginase hypersensitivity.

**Table 2:** HLA alleles associated with asparaginase hypersensitivity

HLA alleles	OR (95% CI)	*p* value	Adjusted OR^#^ (95% CI)	Adjusted *p* value^#^
HLA allele associated with at least one asparaginase hypersensitivity				
B*51:01	1.2 (0.5–3.1)	0.81	NA	NA
HLA alleles associated with l-asparaginase hypersensitivity				
B*46:01	3.8 (1.4–10.1)	< 0.01*	3.5 (1.3–10.5)	0.02*
B*51:01	1.3 (0.5–3.4)	0.81	NA	NA
DRB1*09:01	4.3 (1.6–11.4)	< 0.01*	4.4 (1.6–13.3)	< 0.01*

CI, confidence interval; HLA, human leukocyte antigen; NA, not applicable; and OR, odds ratio

^*^p < 0.05

^#^Adjusted for, age, gender and the diagnosis of B cell acute lymphoblastic leukaemia

This is the first study that explores the association between HLA genotypes and asparaginase hypersensitivity among Han Chinese children. HLA-B*46:01 and DRB1*09:01 were significantly associated with l-asparaginase hypersensitivity. A previous study involving patients with European ancestry showed that HLA-DRB1*07:01 was a high-risk allele associated with asparaginase hypersensitivity [5]. However, a subsequent study did not reproduce the same association in the Asian ancestry, probably due to the smaller Asian sample size [4]. We identified another HLA class II allele, HLA-DRB1*09:01, being associated with l-asparaginase hypersensitivity. HLA class II molecule was implicated in various IgE-mediated allergies, including asthma, food and drug hypersensitivities [6, 7], and different HLA alleles have also been implicated in allergic diseases among different ethnic populations [8]. Our study also demonstrated that HLA-B*46:01, a class I allele, was associated with immediate-type l-asparaginase hypersensitivity. Conventionally, HLA class I alleles were known to be associated with severe cutaneous adverse reactions (SCAR), such as the association between HLA-B*15:02 and carbamazepine-induced SJS [9]. However, a recent study in children and adults has demonstrated the role of allergen-specific CD8 + T cells with IgE-mediated peanut hypersensitivity and was associated with HLA-A*02:01 [10], therefore, indicating the IgE-mediated immediate-type reactions are not limited to be associated with HLA class II alleles, but also class I alleles.

The management of l-asparaginase hypersensitivity varies between centres, mainly depending on the availability of expertise and alternative drugs, including peg-asparaginase and Erwinase. In centres where access to peg-asparaginase and Erwinase is limited, an alternative approach using steroid and anti-histamines as pre-medications followed by desensitisation if needed [11]. Evaluation of polyethylene glycol (PEG) hypersensitivity may be warranted in patients with exclusive peg-asparaginase allergies or a history of allergic reactions to drugs containing PEG as excipients. 80% of our patients with exclusive peg-asparaginase hypersensitivity carry HLA-A*11:01. This group of patients is possibly allergic to the PEG molecule rather than the asparaginases. PEGs are used widely in drugs, cosmetics and laxatives, and hypersensitivity to PEGs can be severe but rare [12]. Patients who are allergic to PEGs usually reacted against PEGs of higher molecular weights and higher concentrations. Skin prick and intradermal skin tests are possible ways to investigate PEGs allergies [12]. The identification and addition of pre-treatment HLA typing may provide a guide for individualised treatment design in choosing asparaginase preparations to minimise the risk of allergic reactions.

This study has to be interpreted with several caveats. First, only patients who had higher risk or relapsed diseases had HLA genotyping performed in anticipation to receive bone marrow transplantation. There is a possibility that the relapsed cases may have a higher chance of developing asparaginase hypersensitivity as they have a higher exposure to asparaginases. Nevertheless, there were no significant differences between our patients with and without asparaginase hypersensitivity in their clinical outcomes. Second, these patients identified as having asparaginase hypersensitivities were based on clinical observations. Additional investigations such as serum tryptase level and drug provocative test were not performed. Nevertheless, these allergic reactions were all being evaluated and clearly documented by their physicians-in-charge. Therefore, the labelling of asparaginase allergy can be considered as reliable.

The identification of ethnic-specific high-risk HLA alleles may help guide choosing which preparations of asparaginase to use and possibly the early involvement of allergists in managing these at-risk patients. A large-scale, prospective, Chinese studies will be warranted to confirm the findings.

## Supplementary Information

**Additional file 1**. The Additional File contains the details of the study methodology and results of the enrichment analysis, including data retrieval, HLA typing, identification of HLA alleles enriched and associated with asparaginase hypersensitivity, and statistical analysis.

## Data Availability

The datasets used and/or analysed during the current study are available from the corresponding author on reasonable request.

## Abbreviations

ALL: Acute lymphoblastic leukaemia
BMT: Bone marrow transplantation
CI: Confidence intervals
HLA: Human leukocyte antigen
IgE: Immunoglobulin E
NHL: Non-Hodgkin lymphoma
OR: Odds ratio
PEG: Polyethylene glycol
