# Supplementary material for: HLA alleles associated with asparaginase hypersensitivity in Chinese children
Source: J Hematol Oncol. 2021 Oct 30;14:182. doi: 10.1186/s13045-021-01201-3 (PMC8557538; doi:10.1186/s13045-021-01201-3)
Supplement: Supplementary file 1 — Additional file 1. The Additional File contains the details of the study methodology and results of the enrichment analysis, including data retrieval, HLA typing, identification of HLA alleles enriched and associated with asparaginase hypersensitivity, and statistical analysis. [file 13045_2021_1201_MOESM1_ESM.docx]

**Study Methodology**

**Data retrieval**

All children in Hong Kong under 18 years old who were diagnosed with any malignancies and were candidates for bone marrow transplantation would have HLA-genotyping performed by the Division of Transplantation and Immunogenetics, Department of Pathology, Queen Mary Hospital in Hong Kong. All children under 18 years old who have HLA-typing performed between 1 January 2009 and 31 December 2019 were retrieved and identified. The prescription records of these patients for L-asparaginase, peg-asparaginase and Erwinase were matched with their identity using the Hospital Authority Clinical Data Analysis and Reporting System (CDARS), a centralized database containing in and out-patient prescription records for all patients managed in the public hospital system. An allergy physician reviewed the clinical records of patients under 18 years old who have received any forms of asparaginases (L-asparaginase, peg-asparaginase and Erwinase). Their demographics and clinical characteristics, including ethnicity, date of birth, gender, primary diagnosis, age at primary diagnosis, HLA typing, presence of asparaginase hypersensitivity, type of asparaginases that they were allergic to, the allergic manifestations (immediate versus delayed, anaphylaxis versus non-anaphylaxis), and clinical outcomes (relapse, need for bone marrow transplantation and death) were recorded. Only Chinese children with the diagnosis of ALL, mixed phenotype acute leukaemia and NHL who have received asparaginase and were HLA-typed were included in the association analysis. We defined anaphylaxis based on the latest World Allergy Organization guideline.^1^ Patients ≥18 years old at their primary diagnosis, non-ethnic Chinese patients, and primary diagnoses other than ALL, mixed phenotype acute leukaemia and non-Hodgkin lymphomas (NHL) were excluded from this study to reduce heterogeneity of the study subjects (Figure 1).

**Human leukocyte antigen (HLA) typing**

HLA genotype was performed using Sanger sequence-based typing before the end of 2018 and next generation sequencing starting 2019 after introducing Illumina MiSeq system platform (San Diego, USA). Sequence-based typing methods utilise the specific primers of the SBTexcellerator® HLA typing Kit (Genome Diagnostics, Utrecht, the Netherlands). Next Generation Sequencing (AllType NGS; One Lambda, Canoga Park, California) on the Illumina MiSeq system platform. The reads were analysed using the TypeStream^TM^ Visual Software version 1.3.0 (One Lambda, Canoga Park, California).

**Identification of HLA alleles enriched and associated with asparaginase hypersensitivity**

The frequency in percentages and the 95% confidence intervals (CI) of each HLA allele were calculated among the included children with or without asparaginase allergy and were compared with the HLA allele frequencies in children without exposure to asparaginase and with the Hong Kong general population.^2^ Published data generated from the Hong Kong Bone Marrow Donor Registry (HKBMDR) was obtained for the mean allele frequencies presented in percentages. When the lower 95% confidence limits of HLA alleles frequencies among asparaginase hypersensitive patients were higher than the mean allele frequencies in the Hong Kong general population, they would be considered high-risk alleles for asparaginase hypersensitivity. To ensure that these high-risk HLA alleles identified were enriched because of asparaginase hypersensitivity, but not due to their primary diseases, the percentages and 95% CI of these HLA alleles were calculated among the asparaginase non-hypersensitive groups and were compared with the frequencies in the Hong Kong general population. When the lower 95% confidence limits of the HLA alleles among the patients who have been exposed but not hypersensitive to asparaginase were not higher than the mean allele frequencies in the Hong Kong general population, the HLA alleles would be considered as genuinely enriched in the asparaginase hypersensitive group. The enriched HLA alleles were analysed by comparing the percentages and 95% CI between the non-exposed group and the Hong Kong general population. HLA typing was performed for non-exposed group for other clinical indications warranting bone marrow transplantation, such as primary immunodeficiency and relapsed neuroblastoma. When the 95% CI of the HLA alleles among the non-exposed group overlaps with the mean allele frequencies in the general population, the HLA alleles distribution would be considered as similar between the non-exposed group and the general population. The HLA alleles identified to be genuinely enriched in the asparaginase hypersensitive group were further analysed by calculating their odds ratios and adjusted for potential confounders to determine their association with asparaginase hypersensitivity.

**Statistical analysis**

Comparison of the demographics, diagnoses and outcomes between the included asparaginase hypersensitive and non-hypersensitive groups were performed using the Mann-Whitney U test and Fisher’s exact test for continuous and categorical variables, respectively. The crude odds ratios of each high-risk allele were further calculated using Fisher’s exact test. Multiple logistic regression model was used to adjust for potential confounders and to estimate the adjusted odds ratio. A p-value of <0.05 was considered statistically significant. All analysis was calculated using Prism® version 9 (San Diego, USA).

**Ethics**

The University of Hong Kong/Hospital Authority Hong Kong West Cluster Institutional Review Board (Reference number: UW 21-208) and the Hong Kong Children’s Hospital Research Ethics Committee (Reference number: HKCH-REC-2021-22) approved the study.

**Patient selection process**

493 patients aged <18 years old had HLA-typing. 30 patients were excluded as they were non-Chinese. 112 of them have received at least one form of asparaginase. Five patients were considered as outliers and were excluded as asparaginase was not commonly used to treat these conditions: two had blastic plasmacytoid dendritic cell neoplasm, one had acute myeloid leukaemia, one had chronic myeloid leukaemia, and one had post-transplant lymphoproliferative disorder. 107 patients were included for the analysis. (Supplementary Figure 1)

**Supplementary Figure 1**

**
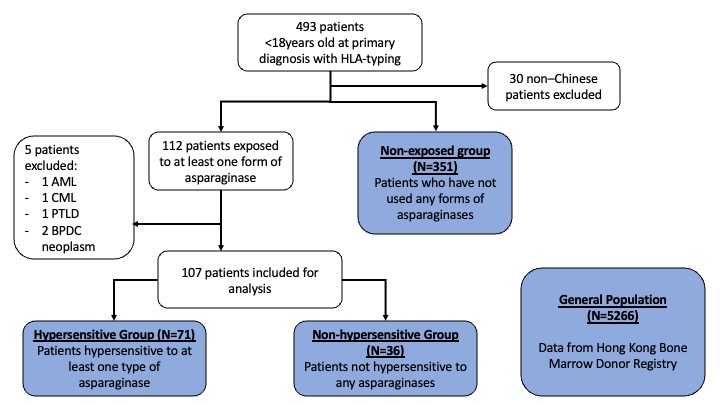
**

**Supplementary Table 1.** Distribution of asparaginase exposure, hypersensitivity and anaphylaxis to L-asparaginase, peg-asparaginase and erwinase.

|  | Hypersensitive (%) | Anaphylaxis (%) |
| --- | --- | --- |
| L-asparaginase (n=105) | 65 (61.9) | 25 (23.8) |
| Peg-asparaginase (n=69) | 38 (55.1) | 25 (36.2) |
| Erwinse (n=37) | 8 (21.6) | 5 (13.5) |

**Footnote:**

6 patients (5.6%) were hypersensitive to all three forms of asparaginases.

**Identification of enriched alleles**

Supplementary Table 2 showed the HLA alleles enriched among our patients with asparaginase hypersensitivities. HLA-B*51:01 was enriched among patients with hypersensitivity to at least one type of asparaginase, present in 16 patients (22.5%, 95% CI 12.8 – 32.3%) and was significantly higher than the mean allele frequency in the general population (7.5%, 95% CI 6.8 – 8.2%). Among patients who were not hypersensitive to asparaginase, seven patients carried B*51:01 (19.4%, 95% CI 6.5 – 32.4%) and the 95% CI was similar to the general population. The frequencies of HLA-B*51:01 was also similar between the non-exposed (9.7%, 95% CI 6.6 – 12.8%) and the general population (7.5%, 95% CI 6.8 – 8.2%). Among patients with L-asparaginase hypersensitivity HLA-B*46:01 (40%, 95% CI 28.1 – 51.9%), B*51:01 (23.1%, 95% CI 12.8 – 33.3%) and DRB1*09:01 (43.1%, 95% CI 31.0 - 55.1%) were considered enriched, as the 95% lower confidence limit of these alleles were significantly higher than the mean allele frequencies in the general population (HLA-B*46:01 27.1%, B*51:01 7.5% and DRB1*09:01 28.4%), and were similar or lower between the L-asparaginase non-hypersensitive group [HLA-B*46:01 (15%, 95% CI 3.9 – 26.1%), B*51:01 (20.0%, 95% CI 7.6 – 32.4%) and DRB1*09:01 (15.0%, 95% CI 3.9 – 26.1%)] and the general population. The 95% CI of HLA-B*46:01, B*51:01 and DRB1*09:01 were all similar between the non-exposed groups and the general population. No HLA alleles were enriched for other subgroups including patients with L-asparginase anaphylaxis, peg-asparaginase anaphylaxis, hypersensitivity to both L-asparaginase and peg-asparaginase, and hypersensitivity to all asparaginases.

**Supplementary Table 2:** HLA alleles enriched in patients hypersensitive to asparaginase comparing with the normal population

| HLA alleles |  | | No. of patients carrying the allele (%) | 95% CI | HKBMDR mean allele frequency (%) |  |
| --- | --- | --- | --- | --- | --- | --- |
| HLA alleles enriched in patients hypersensitivity to at least one asparaginase  (Total number of patients exposed to asparaginase = 108) | | | | | | |
| B*51:01 | Overall (n=458) | | 57 (12.4) | 9.4 – 15.5* | 7.5 |  |
|  |  | Hypersensitive (n=71) | 16 (22.5) | 12.8 - 32.3* |  |  |
|  |  | Non-hypersensitive (n=36) | 7 (19.4) | 6.5 – 32.4 |  |  |
|  |  | Not exposed to any  asparaginase (n=351) | 34 (9.7) | 6.6 – 12.8 |  |  |
| HLA alleles enriched in patients hypersensitivity to L-asparaginase  (Total number of patients exposed to L-asparaginase = 105) | | | | | | |
| B*46:01 | Overall (n=458) | | 143 (31.2) | 27.0 – 35.5 | 27.1 |  |
|  |  | Hypersensitive (n=65) | 26 (40.0) | 28.1 - 51.9* |  |  |
|  |  | Non-hypersensitive (n=40) | 6 (15.0) | 3.9 – 26.1^#^ |  |  |
|  |  | Not exposed to L-  asparaginase (n=353) | 111 (31.4) | 26.6 – 36.3 |  |  |
| B*51:01 | Overall (n=458) | | 57 (12.4) | 9.4 – 15.5 | 7.5 |  |
|  |  | Hypersensitive (n=65) | 15 (23.1) | 12.8 - 33.3* |  |  |
|  |  | Non-hypersensitive (n=40) | 8 (20.0) | 7.6 – 32.4 |  |  |
|  |  | Not exposed to L-  aspargainase (n=353) | 34 (9.6) | 6.6 – 12.7 |  |  |
| DRB1*09:01 | Overall (n=458) | | 156 (34.1) | 29.7 – 38.4 | 28.4 |  |
|  |  | Hypersensitive (n=65) | 28 (43.1) | 31.0 - 55.1* |  |  |
|  |  | Non-hypersensitive (n=40) | 6 (15.0) | 3.9 – 26.1**^#^** |  |  |
|  |  | Not exposed to L-  asparaginase (n=353) | 122 (34.6) | 29.6 – 39.5 |  |  |

*95% lower confidence limit higher than the mean frequency in the normal population

^#^95% upper confidence limit lower than the mean frequency in the normal population

CI – confidence interval; HKBMDR – Hong Kong Bone Marrow Donor Registry; HLA – human leukocyte antigen

**Footnotes:**

1. 105 (98.1%) have received L-asparaginase. One patient with B cell ALL and one patient with T cell ALL did not receive L-asparaginase, as the newer treatment protocol used peg-asparaginase as the first-line treatment. The patient with T cell ALL developed anaphylaxis to peg-asparaginase, but tolerated Erwinase. Both patients never received L-asparaginase
2. No HLA alleles were enriched for patients with L-asparginase anaphylaxis, peg-asparaginase anaphylaxis, hypersensitivity to both L-asparaginase and peg-asparaginase, and hypersensitivity to all asparaginases.

**References**

**1.** Cardona V, Ansotegui IJ, Ebisawa M, et al. World Allergy Organization Anaphylaxis Guidance 2020. *World Allergy Organization Journal.* 2020;13(10):100472. doi:<https://doi.org/10.1016/j.waojou.2020.100472>

**2.** Kwok J, Tang WH, Chu WK, et al. High resolution allele genotyping and haplotype frequencies for NGS based HLA 11 loci of 5266 Hong Kong Chinese bone marrow donors. *Hum Immunol.* 2020;81(10-11):577-579. doi:10.1016/j.humimm.2020.08.005
